# Challenges in the Management of Geriatric Obesity in High Risk Populations

**DOI:** 10.3390/nu8050262

**Published:** 2016-05-04

**Authors:** Kathryn N. Porter Starr, Shelley R. McDonald, Julia A. Weidner, Connie W. Bales

**Affiliations:** 1 Department of Medicine, Duke University Medical Center, P.O. Box 3003, Durham, NC 27710, USA; Shelley.mcdonald@dm.duke.edu (S.R.M.); jaw5516@gmail.com (J.A.W.); connie.bales@dm.duke.edu (C.W.B.); 2 Geriatric Research Education and Clinical Center, Durham VA Medical Center, Durham, NC 27710, USA

**Keywords:** obesity, older adults, frailty

## Abstract

The global prevalence of obesity in the older adult population is growing, an increasing concern in both the developed and developing countries of the world. The study of geriatric obesity and its management is a relatively new area of research, especially pertaining to those with elevated health risks. This review characterizes the state of science for this “fat and frail” population and identifies the many gaps in knowledge where future study is urgently needed. In community dwelling older adults, opportunities to improve both body weight and nutritional status are hampered by inadequate programs to identify and treat obesity, but where support programs exist, there are proven benefits. Nutritional status of the hospitalized older adult should be optimized to overcome the stressors of chronic disease, acute illness, and/or surgery. The least restrictive diets tailored to individual preferences while meeting each patient’s nutritional needs will facilitate the energy required for mobility, respiratory sufficiency, immunocompentence, and wound healing. Complications of care due to obesity in the nursing home setting, especially in those with advanced physical and mental disabilities, are becoming more ubiquitous; in almost all of these situations, weight stability is advocated, as some evidence links weight loss with increased mortality. High quality interdisciplinary studies in a variety of settings are needed to identify standards of care and effective treatments for the most vulnerable obese older adults.

## 1. Overview of the Obesity Challenge in High-Risk Populations

**Figure 1:**
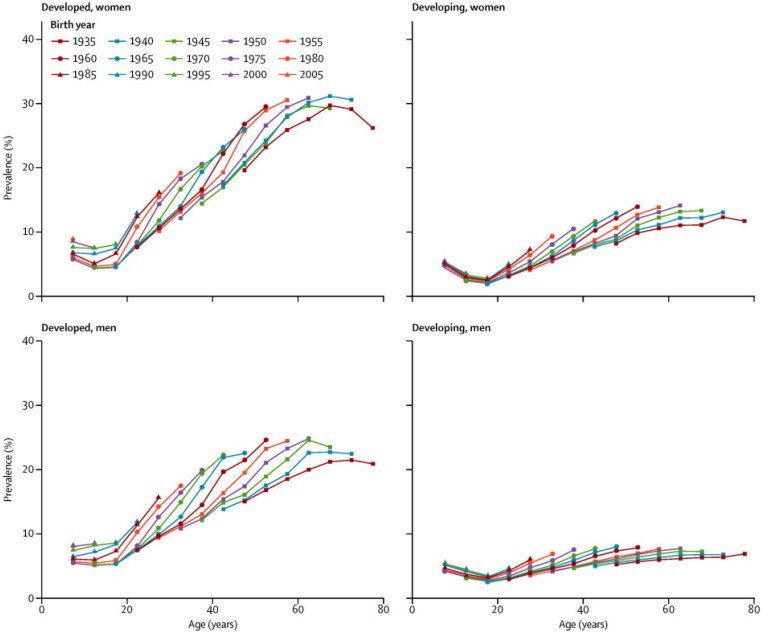
Obesity prevalence for women and men in developed and developing countries presented by age across birth cohorts *. * Findings from the Global Burden of Disease Study 2013 [2], reprinted with permission.

The pervasiveness of the obesity epidemic in the older adult population is poorly recognized on a global scale and certainly under-studied. One third or more of U.S. adults aged 60 years and older have body weights in the obese range (body mass index (BMI) >30 kg/m^2^) [1]; moreover, obesity is increasingly becoming a global health challenge. As illustrated in Figure 1, the findings of the Global Burden of Disease Study 2013 revealed a worldwide increase of 27.5% in prevalence of overweight and obesity between 1980 and 2013. The findings showed that body mass peaked at age 55 years for men (25% obese) and at age 60 years for women (31.3% obese) in developed countries [2]. Age patterns of obesity were similar in developing countries, but with considerably lower prevalence rates; however, in both developed and developing countries successive cohorts seemed to be gaining more weight at all ages. With the phenomenon of “global graying” occurring simultaneously, the stage is set for a global acceleration of diseases and disabilities that are age- and obesity-linked [3].

Obesity and aging are each strong and independent risk factors for metabolic dysfunction, such as impaired glucose intolerance and cardiovascular disease, so the obese older person is especially vulnerable to such metabolic derangements [4,5]. Additionally, this population faces functional limitations that create a cycle of inactivity, further weight gain, and functional deterioration [6]. Importantly, obesity represents a state of malnutrition that does not preclude the coexistence of inadequacies of some important nutrients (e.g., protein, vitamins, minerals). Age-related decreases in calorie requirements make the consumption of essential nutrients a difficult task and consequently, many obese older adults may actually be undernourished. Challenges associated with excess adiposity, including exacerbated chronic disease, increased disability, and decreased quality of life, enhance the on-going risk for malnutrition in this cohort.

The study of geriatric obesity and its management is a relatively new area of research, and this is especially true for groups of obese elders with important additional risk factors for malnutrition, including poverty, isolation, healing challenges due to surgery and/or hospitalization, institutionalization, and advanced mental or physical disabilities. This review focuses on a number of high-risk sub-groups of the “fat and frail” population. After a brief overview of the important issues related to geriatric obesity and the controversies generally associated with weight reduction in older adults, we will explore the state of the science with regards to special populations and highlight areas where future study is urgently recommended and needed.

### The Controversial Nature of Weight Management in Older Adults

The optimal body size at different stages of later life remains a matter of much debate [7]. For this reason, it is important at the outset of this discussion to highlight the recognized age-associated shift upwards in recommended BMI and to emphasize evidence supporting the benefits of weight stability at advanced ages. First, it is important to make the distinction between a BMI in the obese range *versus* one in the “overweight” range with regards to optimal health outcomes. An overweight BMI (25.0 to 29.9 kg/m^2^) is not associated with adverse mortality outcomes in older adults. In fact, being overweight is associated with the lowest mortality across all age groups, and this association is pronounced specifically in the older adults [8]. Thus, there are protective effects of overweight on survival and, unlike for frank obesity, no need to consider efforts to change body weight for older adults with a BMI of 25 to 29.9 kg/m^2^. As for the second point, the importance of weight stability in older adults needs to be emphasized [9,10]; we would add, however, that for older adults whose weights have been stable for some time at a normal BMI, we would not recommend any effort to raise the weight to the overweight range.

In general and historic terms, weight loss interventions have been considered controversial for older individuals, even when there is marked obesity, because of the detrimental consequences associated with weight loss (loss of lean mass and bone mineral density, possible mortality effects) and the potential for inadequate intake of essential nutrients [11,12,13,14,15]. Unintentional weight loss in obese elders has been linked with increased multimorbidity over time [16]. However, there is growing evidence that carefully planned and supervised weight reduction in obese older adults yields clinically important benefits with regard to improving Type 2 diabetes, coronary heart disease, osteoarthritis symptoms, and physical function [17,18,19]. The tension between the need to minimize the negative side effects of weight loss and the many important advantages of reducing excess body weight (such as improved glycemic control, reduced osteoarthritis symptoms, better functional status, improved sleep) is thus evident in the literature [9,20,21].

In particular, the observation of increased survival in heavier adults with inflammatory diseases characterized by wasting (cachexia), such as end stage renal disease and chronic heart failure, the so-called “reverse epidemiology” of obesity or “obesity paradox”, contributes to concerns about the advisability of weight reduction in later life [22]. The paradox is that while obesity is an important risk factor for these conditions, once the disease is present, obesity is actually related to better survival [9]. The obesity paradox has been identified for cancer cachexia, end stage renal disease, and chronic heart failure, and potentially even for Type II diabetes [23,24,25,26]; as discussed subsequently, it may also apply to dementia. Theorized reasons for the increased survival attributed to higher BMIs are diverse, but it could relate, in part, to the availability of larger body stores of both energy (fat) and lean mass, as well as a better overall nutritional state.

The obesity paradox paradigm comes into play for many of high-risk situations discussed here, but not all. Free-living older adults whose health status is relatively stable and whose main problem is simply weighing too much can benefit from guidance in achieving and maintaining a healthier body weight through a judicious, gradual program of diet and exercise. As already noted, a growing body of evidence supports the safety and effectiveness of supervised weight reduction for this cohort. Older adults following calorically restricted diets need more careful monitoring than younger adults; they are at risk of lower intakes of key nutrients, such as vitamin D, iron, calcium and protein [27]. Thus, any weight loss intervention in this population should begin with a thorough diet history so that any inadequacies can be addressed at the outset. It is key that the rate of weight loss be conservative—a maximum of about one pound per week; this can usually be achieved by a modest reduction in daily energy intake of about 500 kcal. The diet should contain about 1.0 g/kg high quality protein/day and a low-dose multivitamin/mineral supplement should be included to ensure that all daily nutrient requirements are met. Caloric restriction should always be accompanied by a physical activity prescription to counteract the tendency to lose lean muscle mass and bone during weight loss. Previous studies in obese older adults have shown that a combination of aerobic, strength, and flexibility training, along with a hypocaloric diet, results in robust weight reduction while preserving lean mass and bone. [19]. However, for the obese, frail, older adult, physical activity must be performed “as tolerated” and is unlikely to provide this protection; the best approach for managing obesity in these individuals remains to be elucidated. New evidence indicating the anabolic advantage of enhancing protein intakes (with or without resistance training) as a means of preserving lean muscle mass and improving physical function during weight reduction may hold promise for this population [28,29].

## 2. Obesity and Nutritional Risk in the Community

### 2.1. Reasons for Concern

The majority of older adults would prefer to age in place, rather than enter an assisted living or other long term care community or facility. This means that the aforementioned global increase in aged cohorts will naturally contribute to a major acceleration in the numbers of community dwelling older adults. In the United States alone, 90% of older adults plan to remain in their home for the next five to ten years [30]. While aging in place offers many advantages, it also brings a number of uncertainties. Many of these elders report that they are unsure of their ability to remain at home due to their financial instability and growing health concerns [30]. There is justifiable reason for their uncertainty, as about 80% of older adults have one or more chronic health conditions. Globally, chronic, non-communicable diseases such as heart disease, cancer, and diabetes account for 87% of the disease burden for adults 60 and older in low-, middle-, and high-income countries [31]. The common risk factors associated with the rise in chronic health conditions in community-dwelling older adults include consumption of energy dense and nutrient poor foods, coupled with decreased physical activity. In other words, a major factor driving the increased prevalence of serious chronic health conditions is the global obesity epidemic [32].

Obesity is, and will continue to be, a costly health concern facing the community-dwelling older adult population and, as already noted, it is no longer limited to developed countries. Of course, obesity itself brings functional and metabolic health risks, as already enumerated. But a particular concern occurs in the paradoxical situation when under-nutrition co-occurs with obesity. Despite being obese, many older adults are malnourished in micronutrients due to poor dietary intake and physiological changes associated with aging [33]. For obvious reasons, this malnutrition is much more difficult to recognize in obese than in underweight elders. Of particular concern are obese, food insecure, community dwelling older adults, whose malnutrition can occur “under the radar” for extended periods of time without recognition. Food insecurity has been associated with an increased risk of health related problems, including obesity [34] and weight-related disability [35]. This phenomenon is not simply a result of sedentary lifestyle and caloric over-consumption, but rather a multitude of risk factors including limited access to healthy, affordable foods [36], few community resources targeted towards older adults [37], environmental barriers to physical activity [38], and cycles of over and under dietary consumption [34,39]. In fact, some of the “younger old” obese adults in the community could be the best candidates for healthy weight reduction interventions emphasizing high nutrient density foods and regular physical activity to improve their metabolic and functional health status, but such interventions are unlikely to occur unless improved obesity screening and intervention strategies can be broadly implemented in the community.

### 2.2. Seeking a Best Course for Obesity Management in the Community

The World Health Organization (WHO) has recognized the growing need for environmental changes that support and encourage healthy behaviors in the community. In 2004, the WHO’s Global Strategy on Diet, Physical Activity, and Health was established to foster preventative interventions and comprehensive polices to reduce the global impact of non-communicable disease, with priority to the most vulnerable populations [40]. Many older adults understand the impact of diet and physical activity on overall health. In the 2015 United States of Aging Survey, a representative sample of over 1600 older adults was collected, over two-thirds of those surveyed agreed that that eating a healthy diet and exercising regularly were essential for maintaining good health [41]. Furthermore, older adults reported a strong interest in learning about ways to maintain their health and attending physical fitness classes tailored to their physical needs [41]. Despite the interest in improving and/or maintaining health, many older adults lack support for these healthy lifestyle behaviors from their community and healthcare providers. For low-income, obese, older adults, this support is essential in order to improve their dietary intakes, prevent continued weight gain, manage current conditions and prevent future obesity-related health conditions. At the community level, the primary goals should be to improve nutritional quality and access to food and create environments that promote consumption of nutrient dense foods and increased physical activity in obese, older adult populations.

### 2.3. Government-Supported Programs Offer Potential Assistance

In the United States, national food and nutrition programs have been established for older adult populations. Specifically, the Older Americans Act Nutrition Program (OAANP) provides congregate and/or home-delivered meals, nutritional screening, and nutrition education and counseling to adults 60 and older. In 2013, the OAANP program provided 219 million meals to 2.6 million older adults [42]. As a result of participating, 73% of congregate meal participants and 81% of home delivered meal participants reported eating healthier diets. Despite these findings, previous studies have shown this population to have high prevalence of obesity [35], obesity-related health problems [43], inappropriate eating behaviors [44], poor nutritional status [45], and food insecurity [39] making this a well suited candidate population for healthy weight maintenance/weight loss interventions. West *et al.* implemented the Diabetes Prevention Program, a 12-week evidence-based weight-loss program, in 15 senior centers using trained lay health educators [46]. Participants in the DPP lost significantly more weight compared to the cognitive behavior control (−3.7 kg *vs*. −0.3 kg; *p* < 0.001). Furthermore, 38% of DPP participants lost ≥5% of their body weight compared with only 5% in the control group who lost the same amount of weight (*p* < 0.001). This study, along with others has shown that improvements in consumption of fruit and vegetables [47], physical activity [48,49], physical function [49], and levels of hemoglobin A1C [50] can positively benefit the health of high risk, vulnerable older adults. Senior centers in the U.S. and community centers worldwide [51] can provide an ideal environment in which to implement evidence-based programs that promote healthy weight maintenance and/or loss, consumption of nutrient dense foods, and safe physical activity.

### 2.4. The Community Environment as a Determinant of Health Behaviors

The community environment where older adults live can play a fundamental role in their access to healthy food choices and ability to maintain optimal levels of physical activity. Understanding the connection between the environment and obesity in this population is essential for slowing the growth of obesity in countries across the globe. In a recent study of 5688 older Americans, Pruchno *et al.* [36] found that those living in neighborhoods with a higher number of fast food restaurants, convenience stores, bars, and small grocery stores were more likely to be obese; no association was found between obesity and neighborhoods with a high density of supermarkets. However, these findings are not likely to be broadly generalizable. For example, findings for 12,595 Japanese older participants from the Aichi Gerontological Evaluation Study revealed a positive relationship between BMI and access to supermarkets [52]. Additionally, obesity was only associated with access to fast food restaurants and convenient stores in older adults who lived alone [52]. Findings from these studies illustrate the importance of accounting for cultural differences and emphasize the acute current lack of information about global determinants of nutritional health. Future studies need to examine interventions tailored to the setting. For example, strategies for improving the food environment for older Americans might include increasing the availability of fruits, vegetables and whole grains at convenience stores in areas without supermarkets, whereas the findings for Japanese elders indicate supermarkets may be the best place to offer weight loss and weight maintenance programs.

The community’s built environment also impacts the level of physical fitness in the older adult population. While physical activity is a key component in maintaining a healthy weight and physical function [53], many older adults live in environments that lack safe ways to move around and be physically active. Obese older adults often experience increased functional impairments related to their weight, making environmental determinants of safe physical activity even more important for them. In a systematic review of 31 qualitative studies across five countries, Moran *et al.* found five environmental themes associated with greater physical activity in older adults; pedestrian infrastructure, safety, access to facilities, aesthetics, and environmental conditions (weather and environmental quality) [54]. Further exploration of environmental determinants of senior health is needed so that policies for planning future community environments for elders can take these factors into account.

In summary, opportunities to improve both body weight and nutritional status in the community are hampered by programs that are inadequate at identifying and treating obesity in older adults who would benefit from intervention. Such programs have the potential to promote better health for these elders on dramatic public health scale. Thus, increased efforts to improve the management of geriatric obesity in the community at all levels, clinical practice, medical research, implementation practices, programmatic policies, and the built environment, are strongly encouraged.

## 3. Obesity Increases the Risk of Hospitalization and Surgery

### 3.1. Reasons for Concern

The accumulation of health problems that stem from obesity-related morbidity often leads to the need for hospitalizations and/or surgical interventions later in life. Chronic diseases related to obesity such as diabetes, coronary heart disease (CHD), congestive heart failure (CHF), atrial fibrillation (A-fib), stroke, cancer, and arthritis undoubtedly increases the lifetime prevalence of a hospital admission for older adults. In the US, four of the five top reasons for hospital admissions for older adults are CHF, coronary atherosclerosis, cardiac dysrhythmias, and acute myocardial infarction [55]. CHD is the leading cause of death worldwide [56] and obesity imparts a significantly increased risk for the development of CHD; in fact, for each incremental 5 kg/m^2^ increase in BMI over normal weight, the hazard ratio was 1.27 (95% CI 1.23–1.31) in a very large pooled analysis using 97 prospective cohort studies [55,57].

Often obesity-related medical conditions require surgical approaches for definitive solutions, and this, also, disproportionately affects individuals in the later stages of their lives. Greater than 70% of cardiothoracic procedures are performed on patients over 65 years of age, and almost 60% of general surgery cases are in older adults [58]. Comorbidities associated with need for surgical treatment or procedures include, but are not limited to, coronary revascularization to treat CHD, ablation techniques for A-fib, gallbladder removal due to cholelithiasis, total knee arthroplasty for degenerative arthritis, and resection of cancers (thyroid, esophageal, gallbladder, colon, renal, breast, ovarian, endometrial, and prostate) [59].

It is well known that older adults have higher rates of postoperative complications [60] and obesity is potentially an additional risk factor for adverse surgical outcomes in older adults. Surgical complications associated with obesity include poor wound healing, risk of infection, increased duration of surgery, and respiratory difficulties. However, the impact of obesity on general health outcomes after surgery has yielded mixed results, often based on the degree of obesity. A number of studies have shown reduced 30-day postoperative mortality or even improved long-term survival in those generally either overweight or with milder levels of obesity, thus supporting the “obesity paradox” [61]. The impact of marked obesity is decidedly detrimental when the BMIs are greater than 40 kg/m^2^ and even more so at BMIs greater than 50 kg/m^2^ [61].

### 3.2. Seeking a Best Course for Obesity Management for Hospitalized Older Adults

Optimal management of comorbid medical conditions is critical during any hospitalization for obese older adults in order to prevent complications that may be mainly caused by obesity. Some of the factors where particular attention should be paid for older hospitalized obese adults include:

1. Mobility. Ambulating can be difficult for older adults with obesity and even more so when they are faced with illness. Early mobilization should be promoted as soon as possible after surgery and for those recovering from medical illness because this helps older adults maintain functional abilities, experience less pain, less delirium, and shorter hospital stays [62,63]. Mobility can also prevent deep vein thrombosis and pulmonary embolus, which are associated with obesity. Early referral to physical therapy is important for facilitating safe mobility and will promote the use of assistive devices or even the use of sling or sit-to-stand patient lifts that are sometimes essential for those with sarcopenic obesity. Any activity may serve to negate the greater risk for accelerated loss of muscle mass in older adults when subjected to bed rest.

2. Respiratory conditions. Obstructive sleep apnea (OSA) is considered a disease of obesity and also increases in prevalence with older age [64,65]. OSA may be prevalent in as much as 41% of those undergoing elective surgery, and the use of a screening tool with high sensitivity, such as the STOP-BANG questionnaire, can help identify those at risk of complications including hypoxemia, pneumonia, respiratory failure, low blood pressure, reduced blood flow to the heart, and A-fib [66]. All patients should be screened so that aggravating factors can be minimized (e.g., over sedation from anesthetic agents, opioid pain medications, and sedative hypnotics) and appropriate interventions can be utilized, such as nocturnal oximetry, semi-upright positioning, and continuous positive airway pressure (CPAP) if tolerated. Obesity can cause reduced chest wall compliance and central obesity increases the pressure on the diaphragm from the intra-abdominal organs, making lying supine more difficult and contributing to respiratory muscle fatigue. In the setting of an acute hospitalization, nothing should be done to cause weight reduction as a means to reduce pulmonary complications and, unfortunately, nothing can be done acutely to improve inherent respiratory function. However, with a few weeks to prepare for an elective surgery, respiratory muscle training can reduce postoperative pulmonary complications by improving respiratory muscle strength before surgery and those gains in respiratory strength are maintained after surgery [67,68].

3. Infectious/wound healing factors. Adipose tissue is very metabolically active and contributes to a chronic inflammatory state that may lead to increased susceptibility to infections [18]. Relating complications of obesity to infections is more difficult because coexisting comorbid conditions such as diabetes are also linked to more infections, but in those with obesity, one meta-analysis did find that those with severe obesity are more likely to develop complications from influenza [69]. This may be due in part to an impaired ability to mount a response, as has been shown by an impaired response to the influenza vaccine [61]. The evidence is limited on causal relationships and mechanisms, but obesity nevertheless is associated with many types of infections, including nosocomial (e.g., pneumonia, catheter-associated bloodstream infections, and *Clostridium difficile* colitis), postoperative surgical site infections (superficial or deep), and skin/soft tissue [70]. Skin infections present from a wide variety of pathogens and can be challenging to identify because of redundant skin folds, limited mobility to assist with the exam, and difficulty differentiating inflammatory changes from infectious ones. It is imperative to carefully examine daily all potential sites where infectious complications may arise and avoid poor glucose control [71,72,73]. One study examined whether or not hyperglycemia and infectious complications could be reduced in those on total parenteral nutrition (TPN) by providing a hypocaloric dextrose TPN, however it failed to prevent hyperglycemia, did not significantly reduce the number of infections, but did create a significant nitrogen deficit [74]. Overall, high protein hypocaloric feeding equally improves clinical outcomes in obese hospital patients when compared to a high protein eucaloric feeding. Therefore, the American Society of Parenteral and Enteral Nutrition established a weak recommendation for hypocaloric, high protein feeding starting at 50%–70% of energy needs and 1.2 g/kg of actual body weight or 2–2.5 g/kg of ideal body weight [75]. Future research is warranted and essential for the development of strong clinical guidelines for hospitalized obese, older adults.

Overall, when older obese individuals are hospitalized either due to illness or surgery, they often present with more coexisting chronic medical conditions due to obesity and have more complications during the course of hospitalization from obesity. While these complications may have been avoided altogether by maintaining a lifelong ideal body weight, the hospitalized older adult needs to have their nutritional status optimized to overcome the stressors of chronic disease, acute illness, and/or surgery. Worldwide, the prevalence of malnutrition is exceedingly high in hospital settings, with 39% of hospitalized older adults affected and an additional 47% at risk for malnutrition [76]. Older obese patients in the hospital are certainly at risk for malnutrition due to the increased nutritional requirements coupled with reduced nutrient utilization related to the underlying cause for their hospitalization, and also due to periods of ordered fasting with no oral intake “NPO” while waiting for surgical intervention or illness that limits intake. Increasing energy intake with preoperative carbohydrate loading up to 2 h before surgery has been shown to improve insulin resistance and reduce the length of hospital stay, with no resulting increase in respiratory complications in normal weight individuals [77,78]. The European Society of Anesthesiology [79] and the Enhanced Recovery after Surgery Society [80] support drinking carbohydrate-rich fluids up to two hours before elective surgery in normal weight and obese adults. Enteral nutrition that is started within 24 h following surgery has been associated with significant reductions in postoperative complications and mortality [81]. The least restrictive diets tailored to individual preferences while meeting each patient’s nutritional needs will facilitate the energy required for mobility, respiratory sufficiency, immunocompentence, and wound healing when older obese adults become hospitalized, and thus, slow or prevent the accumulation of new health problems.

## 4. Obesity in Long Term Care Settings

### Reasons for Concern

While uncertainties about obesity reduction in later life continue to hamper intervention, the fact is that obesity has become a significant concern in long term care (LTC) institutions. The rates of moderate to severe obesity (BMI > 35 kg/m^2^) in nursing homes in the United States have grown by nearly 70%, increasing from 14.7% in 2000 to 23.9% in 2010 [82]. This may be due, at least in part, to the lower average age of LTC admission for obese compared to non-obese individuals (78.5 and 82.5 years, respectively) [83]. Moreover, obesity not only increases the likelihood of nursing home admission, but it markedly compounds the demands of nursing care [83,84,85,86].

The phenomenon of global aging is bringing growing pressure on LTC facilities to admit, care for, and utilize their resources on obese residents, who have more extensive care requirements than their normal weight counterparts. It is not surprising, therefore, that LTC facilities lacking the infrastructure to provide proper care for these residents have, in some cases, refused to admit them [83,87,88,89]. Caring for these patients brings a host of obesity-related challenges, both in terms of the facility (*i.e*., special equipment, extra staffing) and nutritional care planning (e.g., appropriate body weight and caloric intake targets are ill-defined) [90,91]. Clearly, the long-term cost of care is amplified in obese patients, who usually require more health services and spend more days in LTC before death compared to the non-obese elderly individuals [92,93,94].

## 5. Seeking a Best Course for Obesity Management in Long Term Care

While obesity-related problems in the LTC setting are easy to enumerate, finding the best approach for managing them is challenging, to say the least. The list of “pros” and “cons” for weight reduction in later life is long, especially when there is considerable medical complexity. For example, in the case of an acute medical condition, obesity is associated with greater mortality risk following nursing home admission. In contrast, once an initial period of nursing home residence is achieved/endured, having a higher BMI is linked to reduced risk of mortality in nursing home patients [95]. In fact, reverse epidemiology in the elderly obese LTC population is well documented [96]. A meta-analysis of 19,538 individuals in nursing homes and LTC facilities worldwide provides evidence of lower mortality rates for those with an obese BMI compared to those with a normal or underweight BMI [97]. Additionally, it has been globally observed that weight-stable obese nursing home individuals survive longer in the nursing home setting and function at a higher level for a longer duration than non-obese counterparts in several retrospective and prospective studies [96,98,99].

However, the findings on mortality cannot justify completely setting aside the reality of the negative impact of Class II or greater obesity on the health status and quality of life of older LTC residents. Obesity magnifies their symptoms of diabetes, cardiovascular disease and hypertension and complicates osteoarthritis, surgical procedures, and wound healing. The risk of falling is increased [100] and two recent trials have linked abdominal obesity with increased risk of hip fracture [101,102]. There are indirect effects on quality of life as well: Sarcopenic obesity is linked with greater risk of depression [103] and can isolate the resident even within the LTC community. The disproportionate amount of time spent by staff and residents attending to their activities of daily living (ADLs) of obese compared to non-obese LTC residents may effectively decrease their sense of dignity, independence, and overall quality of life, as daily care activities become difficult and exhausting; one case report noted that a bath for an obese resident can take up to 70 minutes to complete [83,90].

What solutions might be considered for the future? To date, we can find no evidence in the literature of studies of intentional weight-loss interventions for obesity in the nursing home setting [104]. Given the strong reverse mortality findings and the fact that weight loss interventions in older adults are generally deemed controversial, this is not surprising [15]. For now, weight-maintenance diets seem the best approach for obesity in LTC. In particular, an isocaloric, but nutrient-rich dietary plan would be advised for those with clearly excessive body weights. Past thinking in LTC dietetics has, understandably, focused on preventing underweight. For example, the widely advocated relaxation of therapeutic diet prescriptions is based on the recognized need for underweight patients to consume more calories and nutrients [105]. Thus, the mainstream thinking in LTC dietetics would also opt for weight maintenance in the obese elderly nursing home population. However, weight maintenance will do little to prevent the strain of extra staffing and equipment requirements for obese patients in LTC care settings. Clearly, “up-stream” solutions will be necessary to stem the growing trend of challenges associated with caring for patients who are obese in LTC. Weight-loss interventions in mid-life age categories (30–50 years) should be strongly considered by obese individuals and may well serve to simultaneously reduce the costs of medical care later in life, improve the quality of life of elderly nursing home residents, and decrease the average length of stay for individuals in nursing homes due to later admit age.

In summary, complications of care due to obesity in the nursing home setting are becoming more ubiquitous as the population increases; however, the steps to be taken in the mitigation of obesity in the LTC setting are unclear. For now, we would advise that for elderly LTC residents with Class II or greater obesity, the recommended course of action is a weight-maintenance diet that is nutrient-rich coupled, when possible, with an appropriate exercise routine.

## 6. Populations with Advanced Physical or Mental Disabilities

An individualized approach to obesity, with careful consideration of health and quality of life priorities, should be taken in all situations of advanced physical or mental impairments. If the body weight is only modestly elevated, weight maintenance rather than reduction would always be advised. In the case of marked obesity (Class II or greater) that is having a direct impact on health and quality of life, there is very little guidance in the literature concerning the best approach to use when obesity is accompanied by advanced disabling conditions. In cases of disease states that are likely to progress to a state of inflammation and/or wasting, such as certain cancers, advanced kidney disease, chronic obstructive pulmonary disease or chronic heart failure, no intentional weight reduction is advised. This aligns with the obesity paradox benefit that is often observed with these conditions. Weight stability is also advocated in situations of late stage dementia and in any terminal illness.

In other slightly less severe situations, the benefit/risk relationship is delicate to navigate and needs to be carefully considered on an individual basis. In the case of osteoporosis, a heavier body weight may be advantageous. While obesity may increase the risk of falling, it is also associated with greater bone density and a lessened likelihood to be injured in a fall or sustain a fracture. However, if the obesity is markedly disabling, a modest reduction in body weight could provide substantial benefits by enabling the individual to be more active (advantageous to muscle as well as bone) and less prone to injury.

With regards to mental impairments, specific situations must be evaluated in terms of quality of life for the individual. For example, the best BMI for individuals with dementia may vary with gender; a recent study in Sweden of BMI and mortality in 11,398 individuals with incident dementia showed that a BMI in the overweight range was associated with the best mortality rates in men but for women a normal BMI was linked with best mortality outcomes. This same study showed a neutral relationship between an obese BMI and mortality in this study population [106]. Findings from the IQUARE study in France indicated an amplification of the obesity paradox in nursing home residents with dementia; the authors advised “extreme caution” about initiating weight loss in nursing homes, especially in those with dementia [107].

In the case of other mental health conditions, the interrelationships with obesity in old age are not well delineated, although more studies are beginning to emerge. Suboptimal quality of life and mental health conditions, including depression, often co-occur with obesity [108] and there is some indication that the mental health-obesity association may be stronger in late life than early or middle adulthood [109]. Jackson *et al.* [110] studied obesity and psychological well-being (quality of life, life satisfaction, and depressive symptoms) in 5056 older (≥50 years) men and women living in England and found that those who were obese showed modestly poorer well-being scores in all three domains and that weight discrimination was widely reported. With regards to cognitive function, however, findings from the Korean Longitudinal Study of Aging in 5125 adults, age 45 or older with normal cognitive function at baseline (Mini Mental State examination) showed that obesity was associated with a lower risk of cognitive decline over the six years of follow-up [111]. These findings illustrate the breadth of considerations relating to mental quality of life and the possibility that obesity can have both beneficial and detrimental influences in older individuals.

As we conclude this section, a point should be made about the autonomy of the older individual regarding their own health behavior decision-making. For cognitively-intact elders without a health care power of attorney, the decision about management of their body size is ultimately an individual one, as with any independent adult. Not all of their decisions will be what might be considered optimal. In a study offering nutritional support to older patients following hospitalization, some patients declined to participate because they were happy with the weight loss they had experienced while hospitalized and wished to continue losing weight even during a medical recovery; these beliefs were “echoed by caregivers and, in some instances, by health care providers” [112]. This illustrates the need for a better understanding of the harm associated with under-nutrition (even when overweight or obese) during times of physiological stress and underscores the need for stronger evidence regarding the optimal body size and composition for elders in various health circumstances.

## 7. Recommendations and Conclusions

The obesity paradox continues to be confirmed in an increasing variety of high-risk situations that commonly precipitate in the life course of older individuals. Thus, for the majority of conditions discussed in this review, weight maintenance rather than weight reduction seems the most prudent course based upon available evidence to date. The most beneficial time to consider obesity reduction is early, as in the relatively healthy community-dwelling elder lacking access to adequate resources promoting healthy weight loss and physical activity or the stable moderate to severely obese individual anticipating elective surgery. The recommendation to remain weight stable in late life applies in most of the other situations considered in this review. This includes those who are acutely or seriously ill, residents of long term care facilities, and any obese older adults with complex physical and/or mental health concerns, for whom the best solution for obesity-related functional and metabolic deterioration awaits further study.
